# Colorimetric sensing of chlorpyrifos through negative feedback inhibition of the catalytic activity of silver phosphate oxygenase nanozymes†

**DOI:** 10.1039/c9ra10719c

**Published:** 2020-04-01

**Authors:** Amisha Kushwaha, Gajendar Singh, Manu Sharma

**Affiliations:** Central University of Gujarat Gandhinagar Gujarat-382030 India manu.sharma@cug.ac.in

## Abstract

Intensive use of organophosphate chlorpyrifos pesticides in farming has become a serious issue due to their harmful effects on living beings. Most fruits, vegetables and soil contain chlorpyrifos, and it cannot be rinsed out completely by water washing. Therefore, a selective and sensitive detection of chlorpyrifos is significant. In the present study, the intriguing oxidase-mimicking activity of Ag_3_PO_4_ nanoparticles (NPs) is explored for the fast and selective detection of chlorpyrifos pesticides. Ag_3_PO_4_ NPs exhibit several advantages, such as great catalytic efficiency, high stability, monodispersity and reusability, over other expensive nanozymes *via* a facile one-step sensing. The size, shape, crystal planes and diffraction patterns of the Ag_3_PO_4_ NPs were observed *via* FESEM and HR-TEM. The surface properties and oxidation states were analyzed *via* XPS technique. Ag_3_PO_4_ NPs possess intrinsic excellent oxidase-mimicking properties against 3,3′,5,5′-tetramethylbezidyne (TMB). When chlorpyrifos and Ag_3_PO_4_ NP nanozymes come in proper orientation proximity, chlorpyrifos is oxidized. The oxidized chlorpyrifos produces sulfide ions and chlorpyrifos oxon. The produced sulfide ions in the reaction system interact with Ag_3_PO_4_ NPs and inhibit their catalytic activity by feedback inhibition. Indeed, neither any catalytic site is left to oxidize TMB nor any blue colour appears. Thus, this feedback inhibition phenomenon senses chlorpyrifos pesticides. The calculated limit of detection (LOD) for the standard chlorpyrifos is ∼9.97 ppm, and the efficacy of the Ag_3_PO_4_ NPs calculated in terms of the *K*_m_ value was found to be 0.15 mM. A real sample analysis was carried out by the standard addition method with two soil samples collected from Pethapur and Chiloda villages.

## Abstract 2

Ag_3_PO_4_ oxygenase nanozymatic activity towards chlorpyrifos sensing.

## Graphical abstract

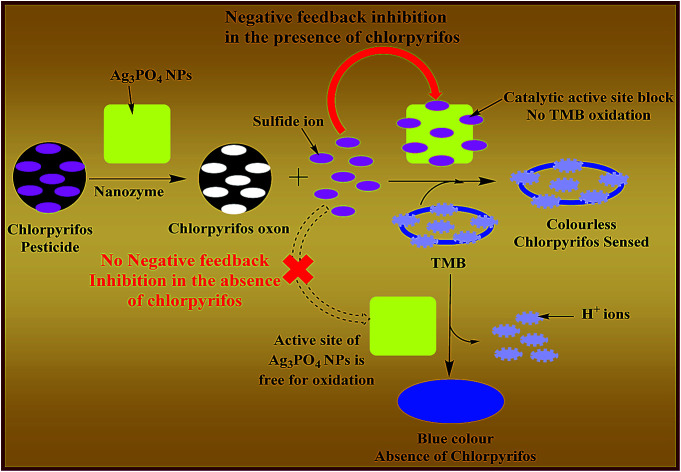

## 1. Introduction

Over the last few decades, the monitoring of hazardous chlorpyrifos in the natural ecosystem has become a serious issue. Pesticides show harmful and adverse effects on human health and on the healthy environment of flora and fauna. Pesticides are used to control undesirable herbs, plants, insects, fungi, nematodes, rodents, fish, bugs, microbes, *etc.* The organophosphate chlorpyrifos is harmful to directly touch, inhale or eat. Its insecticidal action causes the inhibition of the nerve enzyme acetylcholinesterase; consequently, the neurotransmitter acetylcholine accumulates at nerve endings.^1^ Nerve enzyme acetylcholinesterase metabolises the neurotransmitter acetylcholine into choline and acetate in a hydrolytic manner. Chlorpyrifos has a broad spectrum activity that affects the nervous systems of humans, animals and targeted pests by progressively inhibiting acetylcholinesterase, which leads to the loss of sensation.^2^ Exposure to even a small amount can cause tears, runny nose, and increased saliva or drooling symptoms in minutes or an hour. An initial exposure of chlorpyrifos in its complete molecular state form is non-toxic, and as such it moves to all parts of the body. After the oxidation of chlorpyrifos in the body, the toxic chlorpyrifos oxon and sulfide ions are produced.^3^ Then, chlorpyrifos permanently binds acetylcholinesterase and blocks its activity.^4^ Chlorpyrifos is also very toxic to bird species, such as pigeons and grackles, and is moderately toxic to mallard ducks.^4^ When mallard ducks feed on chlorpyrifos, they lay fewer eggs and fewer ducklings are born.^5^ The eggshells also become thinner than normal, and many of the young ducklings die.^6^ Among all birds, robins show the highest mortality from chlorpyrifos exposure. Chlorpyrifos is also very toxic to aquatic invertebrates and fish due to bioaccumulation in their tissues.^7,8^ It is also toxic to honey bees,^9,10^ non-targeted insects and earthworms up to two weeks after it is supplied to soil.^11^ Thus, the selective and sensitive detection of chlorpyrifos is very necessary. Soluble sulfides generally coexist in the state of sulfide ion (S^−^) or hydrogen sulfide (HS^−^), which are corrosive in nature.^12^ Various methods have been developed for the determination of chlorpyrifos, such as electrochemical methods for the determination of chlorpyrifos on a nano-TiO_2_/cellulose acetate composite-modified glassy carbon electrode.^13^ Gold nanoparticles have been applied for the detection of chlorpyrifos in various water samples.^14^ Chlorpyrifos attached to quantum dots shows changes in fluorescence intensity as sensed through flow cytometry.^15^ Surface-enhanced Raman spectroscopy was used for the detection of chlorpyrifos in spinach with silver colloids.^16^ A silver nanohexagon solution is used for the sensing of chlorpyrifos *via* UV-Vis absorption spectroscopy.^17^ These methods are sensitive and specific; however, they involve complex sample preparation, are time-consuming and expensive, and require sophisticated instruments, which limit their further applications. Colorimetric methods are more applicable due to their direct, rapid and simple process of detection. Changes in colour can be observed with naked eye; it does not require expensive or sophisticated instrumentation and can be applied directly in the field.

Recently, several studies have been reported on the colorimetric detection of other biomolecules and toxic ions by exploiting the oxidase-mimicking property. For instance, researchers have used the oxidase-like property of gold nanoclusters for the quantification of melamine in raw milk and milk powder.^18^ Li *et al.* synthesized a cobalt and nitrogen co-doped hierarchically porous carbon hybrid with oxidase mimicking properties for the colorimetric detection of glutathione.^19^ Ag–CoFe_2_O_4_/reduced graphene oxide nanocomposites were engineered for the detection of Hg^2+^.^20^ Researchers found oxidase-like activities in cobalt oxyhydroxide (CoOOH) nanoflakes, which oxidise colourless *o*-phenylenediamine (OPD) to yellow oxidized OPD to sense ascorbic acid.^20^ Verneker *et al.* developed an efficient synthesis of MnFe_2_O_4_*via* a co-precipitation method with controlled morphology; it showed remarkable oxidase mimetic properties.^21^ Graphene quantum dot-Ag nanoparticles show high oxidase and antibacterial properties for Gram-negative, Gram-positive and drug-resistant bacteria.^22^ Cerium oxide was also studied for pH-tuneable oxidase activity for cancer folate biomarker sensors.^23^ Singh *et al.* also studied cerium molybdate-incorporated graphene oxide nanocomposites for glucose sensing.^24^ Researchers also found both peroxidase and oxidase-like activities for AuPt alloy NPs using TMB as a chromogenic material.^25^ Rope-like Co–Fe layered hydroxide nanosheets based on hierarchical structures were studied for intrinsic oxidase-catalytic activity.^26^ Citrate-capped silver nanoparticles exhibit oxidase activity to detect the concentration of mercury ions (II).^27^ Other researchers synthesized Ag@Ag_3_PO_4_ microcubes as oxidase mimics for the ultrasensitive detection of Hg^2+^.^28^

Previous reports show the use of expensive noble metal-based colorimetric sensors based on platinum, rubidium, and gold in their colorimetric sensing systems. All these catalysts are expensive, less stable and not easily available. To overcome these issues, we have designed an efficient Ag_3_PO_4_ NP-based catalyst due to its high oxidizing properties and comparatively low cost. For a long time, Ag_3_PO_4_ NPs have been considered to have antibacterial properties against *Staphylococcus aureus*, *Pseudomonas aeruginosa* and *Escherichia coli*.^29^ Also, it is popularly preferred as a light-sensitive material as it shows excellent photo-oxidative properties for oxygen evolution and photocatalytic dye degradation under visible light irradiation because of its narrow band gap.^30^ Due to these advanced properties of Ag_3_PO_4_ NPs, we were interested to use them for the first time in colorimetric sensors for detection of chlorpyrifos. Many oxidase-mimetic nanomaterial sensors have already proliferated as colorimetric tools with high catalytic properties; however, here, the Ag_3_PO_4_ NPs oxidize TMB in one step with high sensitivity. Therefore, the wide range of practical applications of Ag_3_PO_4_ NPs and their high oxidative properties may offer more importance in the field of sensing.

## 2. Materials and methods

### 2.1 Chemicals and reagents

All chemicals and reagents were used in the entire experiment without further purification. Silver nitrate, di-sodium hydrogen phosphate, 3,3′,5,5′-tetramethylbenzene (TMB), DMSO, sodium acetate, glacial acetic acid, chlorpyrifos, Endosulfan, Fenson, Carbofuran, Aldrin, Dieldrin and Benfuracarb were bought from Sigma-Aldrich India and were used without further purification. Milli-Q water was prepared by an integral water purification system (EMD Millipore).

### 2.2 Synthesis of Ag_3_PO_4_ NPs

**Scheme 1:**
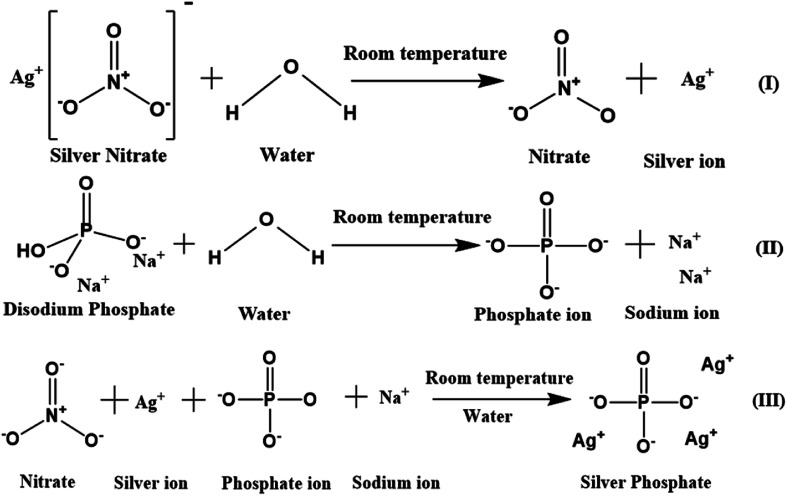
Schematic of silver phosphate nanoparticles synthesized by a simple co-precipitation route.

A simple co-precipitation method was used for the synthesis of Ag_3_PO_4_ NPs.^30^ 60 mM (50 mL) silver nitrate added to Milli-Q water was magnetically stirred at 500 rpm for 15 min in the dark to obtain a transparent solution containing silver and nitrate ions. 20 mM (50 mL) Na_2_HPO_4_ in Milli-Q water was separately stirred for 15 min to obtain a transparent solution containing sodium and phosphate ions. This solution was added dropwise in a continuous manner to the prepared silver nitrate solution, which was then stirred for another 6 h. The obtained lemon yellow precipitate was centrifuged, washed multiple times with Milli-Q water, ethanol and dried at 60 °C (12 h) in the dark. The powdered Ag_3_PO_4_ NPs were stored in the dark for further analysis. A schematic of the mechanism of the Ag_3_PO_4_ NPs synthesis is shown in Scheme 1.

### 2.3 Steady state kinetic assay for the oxidase-like activity of Ag_3_PO_4_ NPs

The oxidase-like activity of the Ag_3_PO_4_ NPs was confirmed by using TMB substrate as a chromogenic material. TMB is converted to the oxidised TMB (oxTMB) blue colour complex in the presence of oxidizing agents such as free radicals of H_2_O_2_, direct sunlight exposure, horseradish peroxidase (HRP) and oxidizing enzymes or nanozymes. The Ag_3_PO_4_ NPs directly oxidize TMB to oxTMB (blue colour) in a one-step process. These nanoparticles act as oxidase nanozymes in the replacement of conventional HRP. Different concentrations of TMB (0.05–0.5 mM) were prepared in DMSO at room temperature. To optimize the reaction system, 60 μL (0.8 mg mL^−1^) Ag_3_PO_4_ NPs were incubated with TMB in 2.0 mL sodium acetate buffer (pH 4.8). The blue oxTMB was analyzed using a UV/Visible spectrophotometer with the maximum absorbance at 652 nm. The obtained data were fitted with the Michaelis–Menten equation and Lineweaver–Burk equation, as given below:1
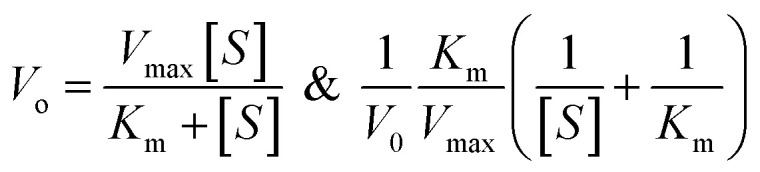
where *V*_0_ is the initial velocity of interaction of the Ag_3_PO_4_ nanozymes with the substrate TMB, *V*_max_ is the maximal velocity of the proposed reaction, [*S*] is the TMB substrate concentration and *K*_m_ is the Michaelis–Menten constant.

### 2.4 Robustness with respect to reusability, pH, temperature and time

For reusability analysis, 80 mL (4.8 pH) acetate buffer, 2.4 mL (0.8 mg mL^−1^) Ag_3_PO_4_ NPs and 4 mL (0.5 mM) TMB were mixed and incubated for 180 s before spectrophotometric analysis. For the next cycle, the sample was centrifuged at 16 000 rpm at 4 °C and re-dispersed in 2.4 mL Milli-Q water; then, we followed the same experiment described above. This process was continued for six repeated cycles. For pH-based robustness, 50 mg of Ag_3_PO_4_ NPs were incubated in each reaction system (pH 2–12) for 1 h. After incubation, the Ag_3_PO_4_ NPs were centrifuged, washed, dried, and subjected to the same sensing method under the pre-optimized conditions described above. For temperature-based stability studies, the NPs were placed in different temperatures (20–80 °C) for 1 h, and the same experiment was performed. For long-term stability studies, the oxidase-mimicking activity of the Ag_3_PO_4_ NPs was checked by recording the absorbance of oxTMB at 652 nm every alternate day up to 26 days.

### 2.5 Colorimetric detection of chlorpyrifos pesticide

For colorimetric detection, all glassware was thoroughly cleaned with Milli-Q water and aquaregia. 200 ppm of chlorpyrifos stock solution was prepared in DMSO and stored for further use. 2 mL volumes of acetate buffer (pH 4.8) containing different concentrations of chlorpyrifos (20–200 ppm) were prepared by maintaining the volume. 60 μL (0.8 mg mL^−1^) of Ag_3_PO_4_ NPs was added to all the glass vials; then, 100 μL of 0.5 mM TMB solution was agitated at room temperature. The obtained blue solution of oxTMB was analyzed by spectrophotometry.

### 2.6 Selectivity analysis

For selectivity analysis, seven different reaction systems were designed using 200 ppm of seven different pesticides (Benfuracarb, Endosulfan, Fenson, Carbofuran, Aldrin, Dieldrin and Chlorpyrifos) prepared in acetate buffer at pH 4.8. 60 μL (0.8 mg mL^−1^) Ag_3_PO_4_ NPs were added to each solution of pesticides and incubated for 180 s. Further, 0.1 mL (0.5 mM) of TMB solution was distributed in each of the seven reaction systems to check the blue colour of oxTMB.

### 2.7 Real sample analysis

Soil samples were collected from two different agriculture sites (Pethapur Village and Chiloda Village), Gandhinagar, Gujarat. Large particles, roots, *etc.* were separated from the soil samples, and the samples were dried (38–40 °C overnight), minced properly and filtered with clean cotton cloth to obtain homogeneous soil samples. The soil samples collected from Pethapur Village and Chiloda Village were labelled as S1 and S2, respectively. 10 g of each soil sample was agitated, washed at room temperature with 40 mL Milli-Q water, and filtered (Whatman No. 2 filter paper). The same process was repeated with hot (75–80 °C) 40 mL Milli-Q water in continuation, and the settled soil was collected for drying. This washed and dried sample was washed again with 40 mL of DMSO; finally, the filtrate was collected and termed as S1L or S2L. Further, the standard addition method was employed for the colorimetric detection of chlorpyrifos by the Ag_3_PO_4_ NPs catalyst.

### 2.8 Thermocol imprint method

A 4 × 2 (*l* × *b*) cm^2^ rectangle of thermocol was imprinted with two alphabetic letters “A” beside each other. The imprinted letters were filled with 1 mg mL^−1^ Ag_3_PO_4_ NPs; then, the catalyst was maintained inside the imprint overnight and stored as a kit for further use. The thermocol imprint was filled with 4.8 pH acetate buffer, chlorpyrifos and 0.5 mM TMB solution, whereas the other imprint was filled only with acetate buffer and 0.5 mM TMB solution. After 3 min, blue colour was observed in the absence of chlorpyrifos, and no colour was observed in the imprint to which chlorpyrifos was added.

## 3 Characterization

Powder X-ray diffraction (PXRD) patterns were recorded using a Panalytical X Pert Pro (Cu Kα, *λ* = 1.5406 Å, 40 mA, 40 kV) with a step size of 0.03 at the rate of 0.6 per second. The crystallite sizes of the nanoparticles were calculated using the Scherrer equation:2
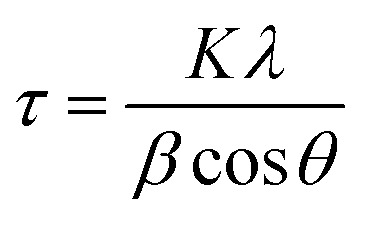
where *τ* is the crystallite size, *K* is the shape factor, and *λ* is the X-ray wavelength. *β* is the corrected line broadening at half maximum intensity, and *θ* is the Bragg angle at the peak position. The UV-Vis absorbance was measured with a Shimadzu UV-1800. Fourier transform infrared spectra were recorded with a PerkinElmer Spectrum 65 instrument. A field emission scanning electron microscope (FE-SEM, Quanta 200 FEG) was used for the morphological analysis. A transmission electron microscope (TEM, Tecnai F20) was employed for the particle size analysis. Differential scanning calorimetry (DSC 6000, PerkinElmer) was used for the thermal stability-based exothermic and endothermic processes. FT-Raman spectroscopy (multi RAM, standalone model) was used to determine the structural and vibrational properties in the 4000–50 cm^−1^ range with a ND: YAG laser source of 1064 nm. XPS (ESCA, Omnicorn Germany) was performed to determine the surface properties of the Ag_3_PO_4_ NPs.

## 4 Results and discussion

**Fig. 1:**
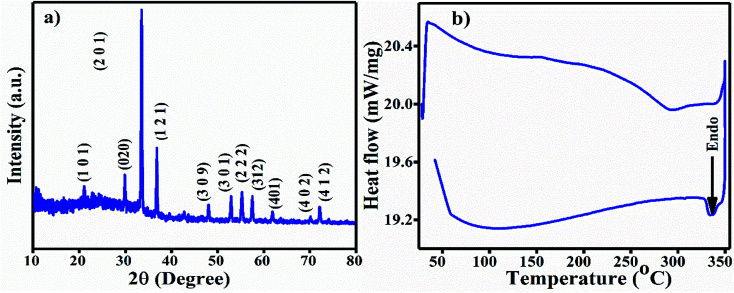
(a) XRD diffraction pattern of the Ag_3_PO_4_ NPs. (b) DSC curve of the Ag_3_PO_4_ NPs.

The XRD pattern of the Ag_3_PO_4_ NPs is shown in Fig. 1(a). All the diffraction peaks show *hkl* values of (101), (020), (201), (121), (309), (301), (222), (312), (401), (402) and (412), corresponding to the 2*θ* values 21.08, 29.91, 33.54, 36.42, 48.02, 52.94, 55.27, 57.48, 61.90, 70.12 and 72.09, which can be indexed to the cubic phase of Ag_3_PO_4_. The calculated crystallite size using Scherrer's formula *τ* = *kλ*/*β* cos *θ* is ∼2.8 nm.^30^ UV-Vis spectroscopy was performed in the wavelength range of 200 to 800 nm using Milli-Q water as the solvent. A broad hump was observed in the visible range, and 3 absorption peaks at 214 nm, 230 nm (π–π*) and 275 nm (*n*–π*) near the UV light absorption range can be observed in Fig. S1a.†

For the FT-IR analysis, ∼2 mg Ag_3_PO_4_ NPs was mixed with 200 mg moistureless KBr, ground well and pressed with a hydraulic press to prepare a pellet. First, the KBr pellet was used for the background; then, the sample was analyzed in the range of 4000–400 cm^−1^ (Fig. S1b†). The two broad peaks originating at ∼3223 cm^−1^ and 1659 cm^−1^ are attributed to the O–H stretching vibration and bending vibration of H–O–H in residual water molecules.^31^ A strong transmittance band as observed at 994 cm^−1^ due to the P–O stretching vibrations of phosphate (PO_4_^3−^). The vibration at 548 cm^−1^ is due to O=P–O group bending vibrations.^32^ All the FT-IR vibration bands are tabulated in Table 1. The differential scanning calorimetric (DSC) curve shows the existence of a small endothermic peak at 334 °C, which may be due to decomposition of the Ag_3_PO_4_ NPs, as shown in Fig. 1b.

**Table 1:** FTIR band positions of the Ag_3_PO_4_ NPs

S. no.	Band position (cm^−1^)	Functional group vibration/bending modes	Reference
1	3223	O–H stretching	31
2	1659	H–O–H bending	31
3	994	P–O stretching	32
4	548	O=P–O bending	32

**Fig. 2:**
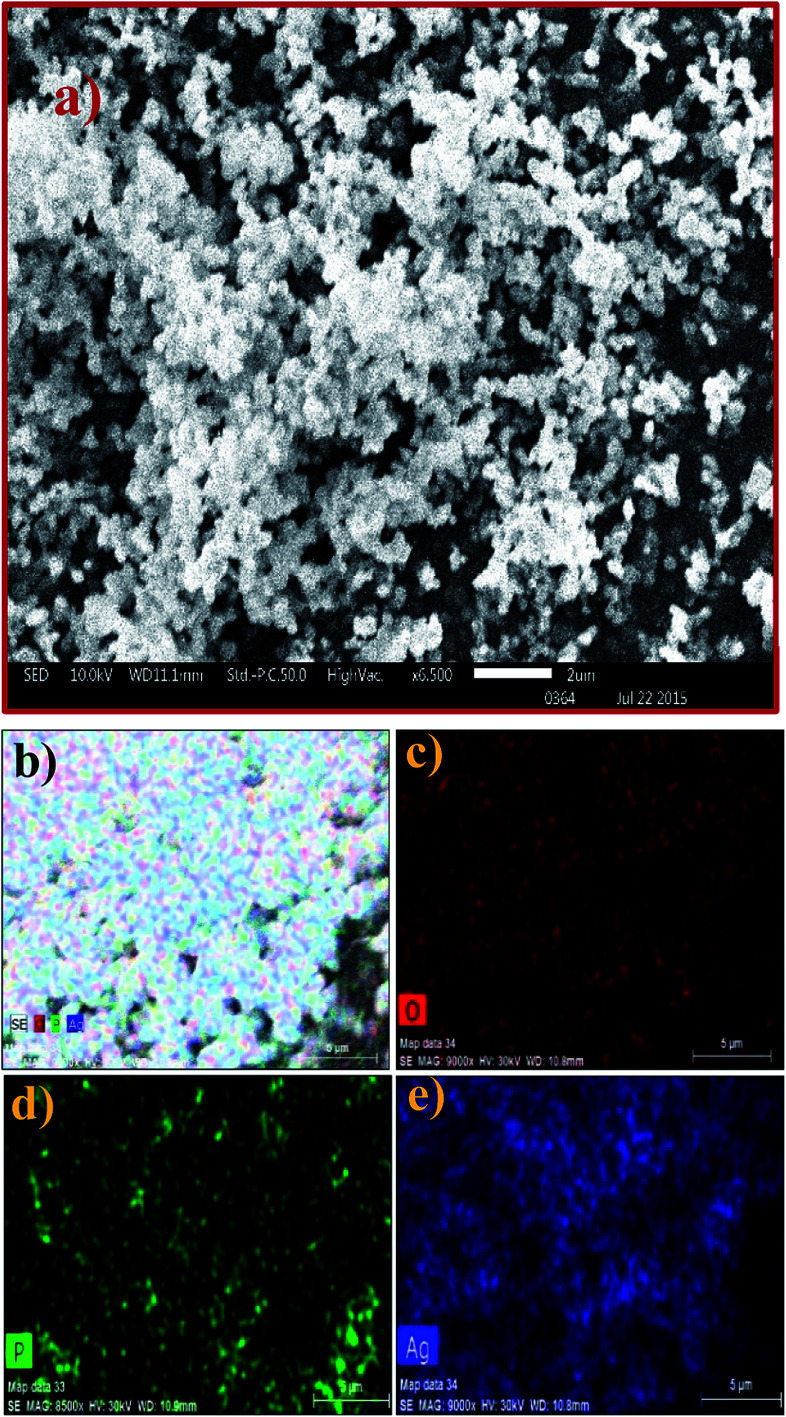
(a) SEM image and (b–e) elemental mapping of the Ag_3_PO_4_ NPs.

The SEM image of the Ag_3_PO_4_ NPs shows aggregated spherical particles with a size range of ∼100 to 200 nm, as shown in Fig. 2a. SEM elemental mapping micrographs clearly show the presence of O, P and Ag without any impurities (Fig. 2b–e).

**Fig. 3:**
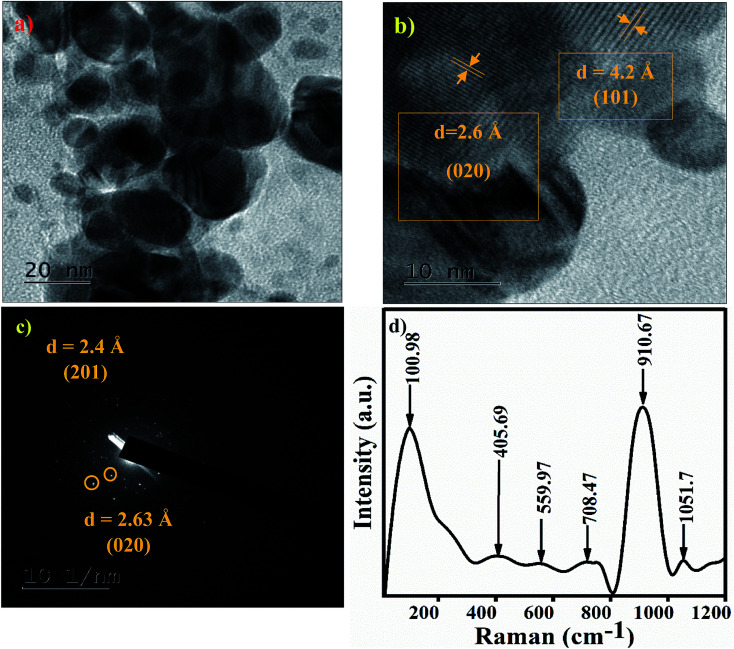
(a) TEM image of the Ag_3_PO_4_ NPs. (b) HRTEM image of the Ag_3_PO_4_ NPs. (c) Electron diffraction pattern of the Ag_3_PO_4_ NPs indexed with the (101) and (020) planes. (d) Raman spectra of the Ag_3_PO_4_ NPs.

The TEM image of the Ag_3_PO_4_ NPs shows spherical particles in the range of 15–40 nm, as shown in Fig. 3(a). The high resolution TEM image of Ag_3_PO_4_ NPs shows the crystal planes (101) and (020), corresponding to the cubic phase of Ag_3_PO_4_ NPs (Fig. 3b). The electron diffraction pattern of the Ag_3_PO_4_ NPs in Fig. 3c shows a bright spot corresponding to the planes (201) and (020). The Raman spectrum of the Ag_3_PO_4_ NPs shows an inelastic, scattered intense peak at ∼910 cm^−1^, attributed to the terminal oxygen vibrational stretching of PO_4_ groups (Fig. 3d). The peak at 1051 cm^−1^ represents asymmetric stretching vibrations of O–P–O [PO_4_], the peak at ∼708 cm^−1^ describes symmetric stretching of the O–P–O bond, the very weak peak at ∼559 cm^−1^ is associated with the asymmetric stretch of P–O–P vibrational bending,^33^ the peak at ∼405 cm^−1^ corresponds to symmetric bending vibration modes related to [PO_4_] clusters, and the peak at 101 cm^−1^ is due to symmetric vibrational bending of Ag–O bonds.^34^

**Fig. 4:**
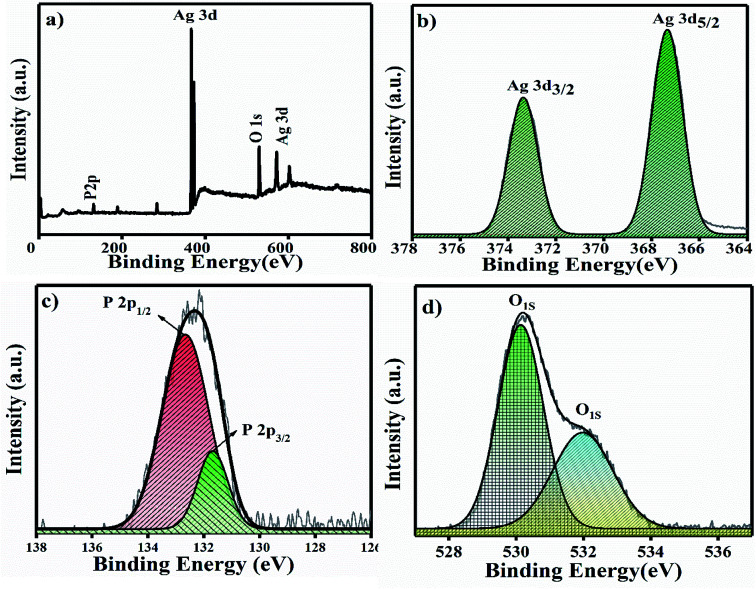
(a) XPS full survey spectra of Ag_3_PO_4_ NPs and (b–d) high resolution spectra of the Ag 3d, P 2p and O 1s spin orbit core levels.

The XPS full survey scan of Ag_3_PO_4_ is shown in Fig. 4a, in which the P 2p, Ag 3d and O 1s spin orbit core levels were detected. High-resolution XPS spectra of the P 2p, Ag 3d and O 1s spin orbit core levels are shown in Fig. 4b–d. Ag 3d has a core level spectrum with two binding energy peaks at 373.34 eV and 367.35 eV due to the electron orbitals of Ag 3d_3/2_ and Ag 3d_5/2_, consistent with the oxidation state of Ag^+^ in Ag_3_PO_4_.^35^Fig. 4c shows the high resolution XPS spectra for the P 2p spin orbit core levels; the two binding energy peaks originating at 132.6 and 131.7 eV correspond to the electron orbitals of P 2p_1/2_ and P 2p_3/2_, which supports that phosphorous is in the P^+5^ oxidation state in Ag_3_PO_4_.^36^

### 4.1 Optimization of TMB and steady state kinetic assay

**Fig. 5:**
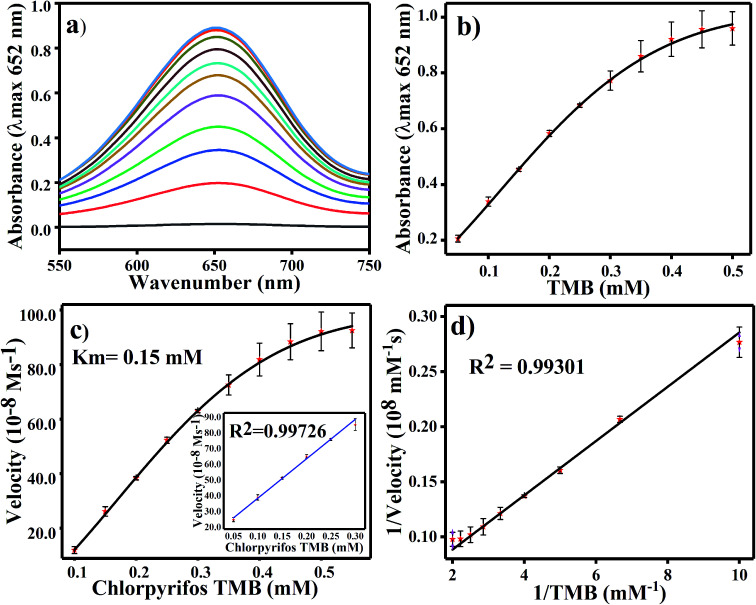
(a) UV-Vis spectroscopy results for various TMB concentrations (0.05–0.5 mM). (b) Linear absorbance graph of TMB at concentrations of 0.05 to 0.5 mM. (c) Kinetic assay analysis of the Ag_3_PO_4_ NPs by Michaelis–Menten plot with the calculated *K*_m_ value and (d) Lineweaver–Burk plot of the Ag_3_PO_4_ NPs.

For TMB optimization, various concentrations of TMB (0.05 to 0.5 mM) were added to 2 mL of acetate buffer (pH 4.8) containing 60 μL of a 0.8 mg mL^−1^ concentration of Ag_3_PO_4_. The UV/Vis results are shown in Fig. 5. As shown in Fig. 5a, as the concentration of TMB increases, the absorbance at 652 increases. The change in absorbance at 652 nm is shown in Fig. 5b, in which the graph plateaus at ∼0.5 mM. 0.5 mM TMB concentration is considered to be the optimal concentration for further experiments. For the steady state kinetic assay, TMB was used as a substrate and chromogenic material. Using the absorption coefficient of TMB (*ε* = 39 000 M^−1^ cm^−1^), a Michaelis–Menten plot was obtained for increasing concentrations of TMB (Fig. 5c). The inset of Fig. 5c shows the linear response of TMB oxidation at all optimal conditions. A double reciprocal or Lineweaver–Burk plot was obtained and is given in Fig. 5d. The Michaelis–Menten constant (*K*_m_) was calculated from the Michaelis–Menten plot and was found to be 0.15 mM.

### 4.2 Effects of Ag_3_PO_4_ concentration, incubation pH, time and temperature

**Fig. 6:**
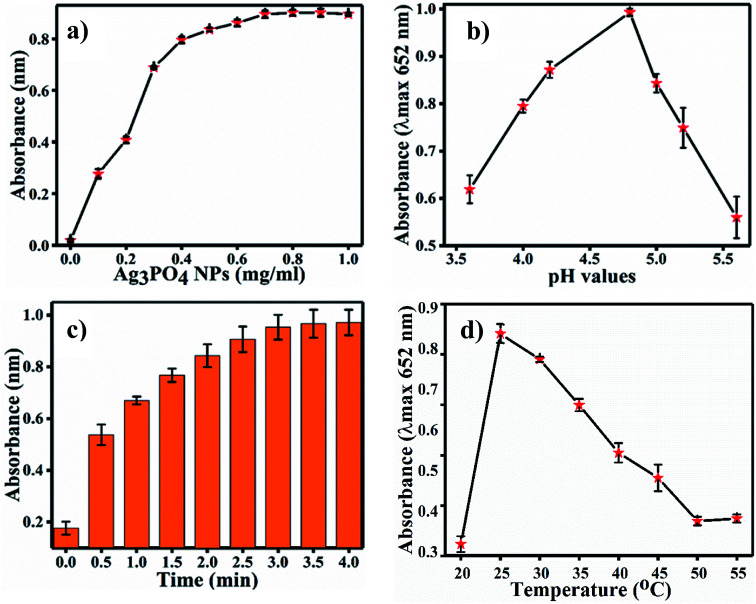
(a) Changes in maximum absorbance with varying concentrations of Ag_3_PO_4_. (b) Calibration plot of pH (c), calibration plot of time in min and (d) calibration plot of temperature at *λ*_max_ = 652 nm for further experiments.

For the optimization of the concentration of Ag_3_PO_4_ NPs, incubation pH, time and temperature, the reaction system was incubated with different concentrations of Ag_3_PO_4_ NPs (0.1–1 mg mL^−1^), pH values (3.6–5.6), times (0–4.0 min.) and temperatures (20–55 °C). Fig. 6a shows the effects of NPs concentration on TMB oxidation. As the NPs concentration increases, the absorbance at 652 increases; the graph plateaus at 0.8 mg mL^−1^ concentration, as shown in Fig. 6a. Hence, 0.8 mg mL^−1^ concentration of Ag_3_PO_4_ was considered as the optimal concentration for further sensing applications. Fig. 6b–d show the effects of incubation pH, time and temperature on TMB oxidation, respectively. The maximum absorbance at 652 nm of oxTMB was recorded at pH 4.8, 3 min and 25 °C. Hence, these parameters were set as the optimal conditions for sensing.

### 4.3 Mechanism of chlorpyrifos sensing

**Scheme 2:**
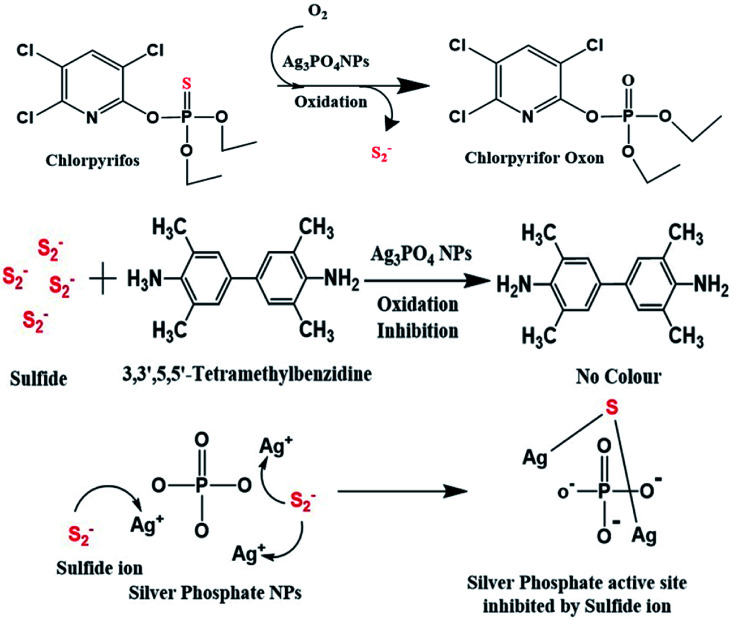
Schematic of the catalytic action of silver phosphate NPs on chlorpyrifos.

As shown in Scheme 2, when the Ag_3_PO_4_ NPs come in proper orientation proximity with TMB, TMB acts as a substrate for the Ag_3_PO_4_ nanozyme. After oxidation, TMB shows a blue colour, which can be tuned to optimal parameters.^37^

Therefore, when chlorpyrifos pesticide comes in proximity with the Ag_3_PO_4_ NPs, it is oxidized to form chlorpyrifos oxon and sulfide ion, as shown in Scheme 2. These produced sulfide ions feedback to the Ag_3_PO_4_ NPs and block their activity. Similarly, W. Qin *et al.* synthesized Co_3_O_4_ NPs for the sensing of sulphite in food and inhibited their colour formation;^38^ also, R. Chen *et al.* created a hydrogen sulfide gas sensor in the presence of silver NPs film.^39^ The sulfide ion released in this reaction after the oxidation of chlorpyrifos is attached to the active site of the Ag_3_PO_4_ NPs, as shown in Scheme 2.

### 4.4 Robustness

**Fig. 7:**
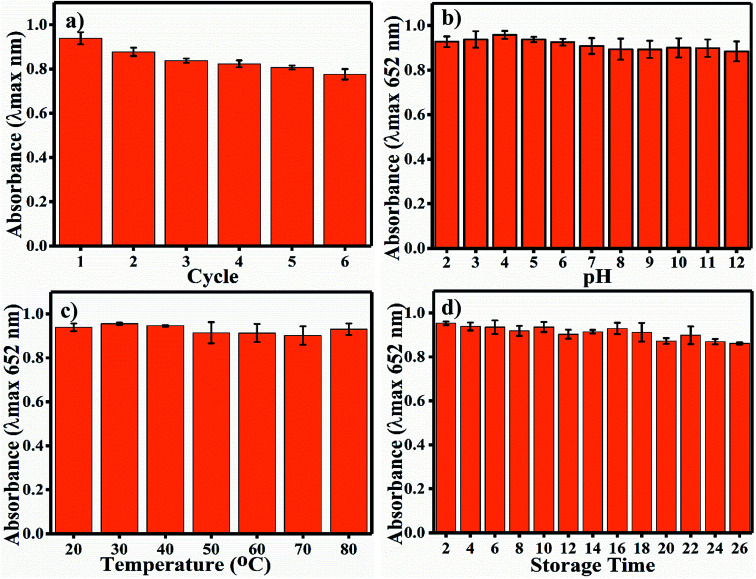
(a) Reusability assay of the Ag_3_PO_4_ NPs, (b) pH stability of the Ag_3_PO_4_ NPs, (c) temperature stability of the Ag_3_PO_4_ NPs. (d) Long-term stability of the Ag_3_PO_4_ NPs after storing the sample at room temperature.

Long-term durability of a nano-sensor is one of the most important aspects required for its satisfactory application. Indeed, the activity of colorimetric nano-sensors depends upon the colour signals generated without substrate and in the presence of the desired sensing materials. Thus, the deviation in colour intensity enables the quantification of the sensing material, and it should not deviate in response to common factors such as pH, temperature and time; also, the reusability of nano-sensors is important. The recyclability of the Ag_3_PO_4_ NPs indicates the potential to reuse the same nanoparticles six times (Fig. 7a). A much less steep decrease in blue colour (oxTMB) was observed up to the sixth cycle. Other nanoparticles have also showed reusable properties, such as Fe^3+^-doped mesoporous carbon nanospheres.^40^ Samples were incubated at different pH values from 2 to 12 for 1 h. The Ag_3_PO_4_ NPs show stability under different harsh pH conditions (Fig. 7b). The catalytic activity is almost constant, and there is no recordable decrease in activity. The temperature stability of the Ag_3_PO_4_ NPs was also recorded in the range of 20–80 °C; as shown in Fig. 7c, no decrease in the reaction activity of the NPs was observed. Similarly, the stability of the NPs was examined during storage from 2 to 26 days every alternative day (Fig. 7d). The storage time reaction stability was also found to be acceptable up to 26 days of continuous incubation. No considerable decrease in the catalytic activity of the NPs was observed. Thus, it can be concluded that the synthesized NPs are stable with respect to pH, temperature, and storage time and can be reused up to six times.

### 4.5 Colorimetric sensing of chlorpyrifos

**Fig. 8:**
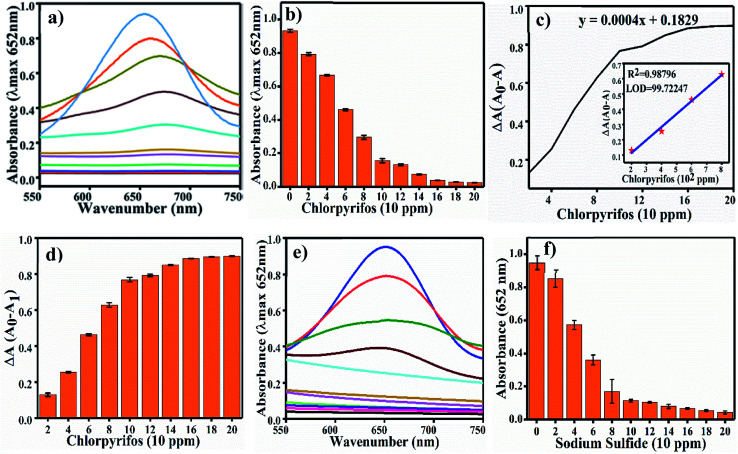
(a) UV-Vis spectra of chlorpyrifos detection. (b) Different concentrations of chlorpyrifos absorbance at 652 nm, (c) Δ*A versus* concentration graph and (d) Δ*A* of different concentrations of chlorpyrifos. (e and f) UV-Vis spectra and sulfide ion sensing.

Colorimetric sensing of chlorpyrifos was studied using TMB, which is a chromogenic substrate for Ag_3_PO_4_ NPs. In the presence of Ag_3_PO_4_ NPs, increasing concentrations of chlorpyrifos decreased the intensity of the peak of the blue colour (oxTMB) (Fig. 8a). 200 ppm chlorpyrifos shows a less intense peak at 650 nm. Further increasing the concentration of chlorpyrifos diminished the blue colour peak. Oxidized chlorpyrifos was converted into chlorpyrifos oxon and sulfide ion, as shown in Scheme 2. Thus, the Ag_3_PO_4_ NPs are confirmed to be oxidase mimetic nanozymes. In Scheme 2, one of the products, sulfide ion, interacts with the Ag_3_PO_4_ NPs; it inhibits the active sites and blocks the oxidizing activity to convert TMB to blue oxTMB as a negative feedback loop. The study of the reaction kinetics measured the reaction rate and affinity of the Ag_3_PO_4_ nanoenzymes towards TMB (substrate).

#### Sulfide ion sensing as a proof of concept experiment

**Fig. 9:**
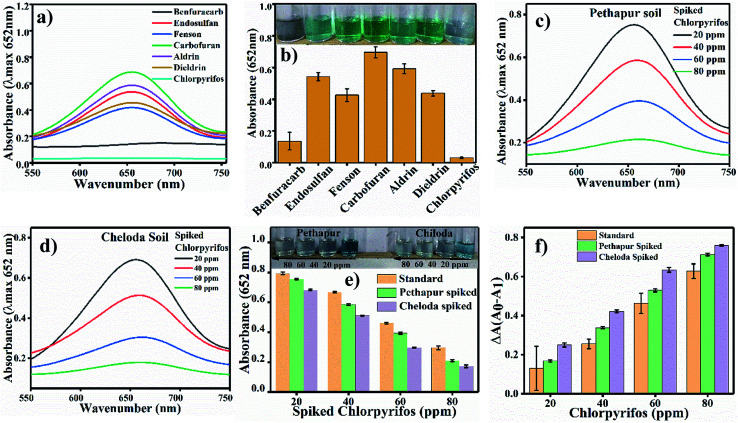
(a) Selective sensing of pesticide with six other pesticides. The oxTMB maximum absorbance appears at 652 nm with non-oxidised TMB. (b) Bar diagram for the selectivity of the absorption of chlorpyrifos with a digital photograph of the colorimetric detection and selection. (c) Pethapur soil spiked sample, (d) Chiloda soil spiked sample and (e) comparative graph of the standard chlorpyrifos and Pethapur and Chiloda-spiked soil samples. (f) Δ*A* graph of chlorpyrifos.

We performed the S^2−^ ions sensing experiment in pre-optimized conditions as a proof of concept. For this experiment, we used Na_2_S with different concentrations (0 ppm to 200 ppm) in water and tested for S^2−^ ions in the reaction solution. Fig. 8e shows the UV/Vis. spectra with increasing concentrations of S^2−^ ions (Na_2_S), and Fig. 8f shows the change in absorbance at 652 nm. As the concentration of S^2−^ increases, the absorbance at 652 nm decreases gradually; this indicates that S^2−^ inhibits the oxidase mimicking activity of Ag_3_PO_4_. For the selectivity test, chlorpyrifos was tested with six other pesticides, namely Benfuracarb, Endosulfan, Fenson, Carbofuran, Aldrin and Dieldrin (their structures are shown in Fig. S2†). A total of seven reaction systems were developed to evaluate potential interference with the Ag_3_PO_4_ NPs in comparison to chlorpyrifos. In order to verify the feasibility of our approach for the detection of chlorpyrifos, the selectivity test results are shown in Fig. 9a and b. At *λ*_max_ = 652 nm for oxTMB, the various pesticides other than chlorpyrifos showed comparatively high colour intensity. The efficacy of the substrate for any enzyme can be estimated by measuring the *K*_m_ value. Therefore, the *K*_m_ value of the Ag_3_PO_4_ NPs calculated for the TMB substrate is low, showing the higher proficiency of the reaction toward the TMB substrate.

The *K*_m_ value (Michaelis–Menten constant) represents the affinity of the catalytic efficiency of an enzyme for a substrate. Many researchers have developed oxidase mimetic nanoparticles, which are tabulated in Table 2. In this table, we can see that the standard enzyme HRP is most traditionally involved in experiments of oxidation based on amperometric biosensors for polyphenol determination.^41^ HRP is used as a glucose biosensor, as established through electro-enzyme catalyst oxidation of glucose^42^ and immobilizing HRP for oxidative polymerization of 2,6-dimethylphenol as a biocatalyst.^43^ Thus, from Table 2, the *K*_m_ value for HRP is about 0.434 mM, and the *K*_m_ value for the Ag_3_PO_4_ NPs is about 0.15 mM.

**Table 2:** Comparative *K*_m_ values of oxidase mimic nanoparticles

S.·no.	Enzyme	Substrate	*K*_m_ (mM)	Reference
1	Ru NPs	TMB	0.234	44
2	HRP	TMB	0.434	45
3	CoFe_2_O_4_	TBM	0.387	46
4	Pt NPs	TMB	0.096	47
5	Ag@Ag_3_PO_4_ MCs	TMB	0.11	28
6	Prussian blue-modified γ-Fe_2_O_3_ magnetic NPs	TMB	0.307	48
7	MnO_2_ NPs	TMB	0.025	49
8	Ag_3_PO_4_ NPs	TMB	0.15	This work

The developed analysis method, which is selective and sensitive for the standard chlorpyrifos, was applied for real sample analysis. Soil samples from Pethapur and Chiloda villages extracted in DMSO show absorbance at 652 nm, as shown in Fig. 9(c) and (d), respectively. The gradient decreases (Fig. 9e) in the absorbance of the standard chlorpyrifos with increasing concentrations of Pethapur and Chiloda DMSO soil extracts, which indicates that chlorpyrifos was sensed (Fig. 9f). The resulting amounts found in the soil extracts, recovery percentages, and RSD values are tabulated in Table 3.

**Table 3:** Analysis of real samples collected from Pethapur and Chiloda villages

Sample	Spiked (ppm)	Detected (ppm)	Recovery (%)	RSD (%) (*n* = 3)
Pethapur village soil sample	20	24.15388	120.7694	5.050763
	40	52.56967	131.4242	1.480893
	60	69.11767	115.1961	2.492734
	80	89.76843	112.2105	2.953513
Chiloda village soil sample	20	35.87434	179.3717	2.896497
	40	65.61423	164.0356	1.717815
	60	82.90019	138.167	1.077412
	80	95.73903	119.6738	2.40221

**Fig. 10:**
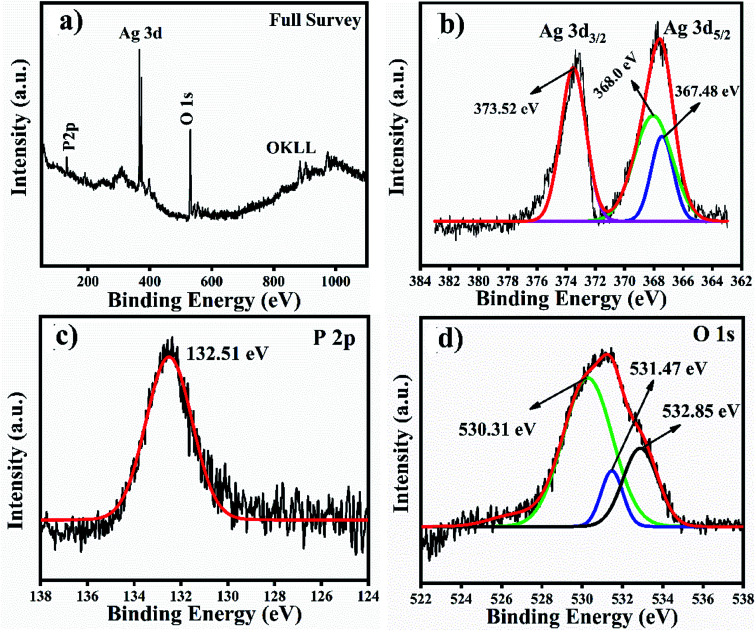
XPS full survey scan and high resolution spectra of Ag, P and O spin orbit core-levels. (a) Full survey scan. (b–d) High resolution spectra of Ag 3d, P 2p and O 1s spin orbit core-levels.

We analysed the Ag_3_PO_4_ catalyst after reacting it with TMB through XPS for Ag valence identification, and the XPS results are shown in Fig. 10. These results have also been added with the manuscript and highlighted with redcolour. According to the XPS results of Ag_3_PO_4_, the OKLL peak can be clearly observed on the surface, showing a higher concentration of oxygen due to exposure to ambient atmospheric conditions. The total scan spectrum binding energy range of 0–1100 eV revealed that Ag, P and O elements coexist in the Ag_3_PO_4_ nanoparticles. Noise peaks were observed after the reaction. In Fig. 10(a), a full survey scan shows the same number of elements which were present before the reaction. In Fig. 10(b), the three peaks positioned at 373.52 eV, 368.00 eV and 367.48 eV in the high resolution spectrum of Ag 3d can be assigned to the electron orbitals of Ag 3d_3/2_ and Ag 3d_5/2_ of Ag^+^, respectively. Thus, after the oxidation process of the Ag_3_PO_4_ NPs, no remarkable change is observed. Therefore, silver is only present in the Ag^+^ state before and after the reaction. In Fig. 10(c), the peak with a binding energy of 132.51 eV is ascribed to P 2p of PO_4_^3−^. The binding energy de-convoluted peaks at 530.31 eV, 531.47 eV and 532.85 eV (Fig. 10d) were assigned to O 1s.

**Fig. 11:**
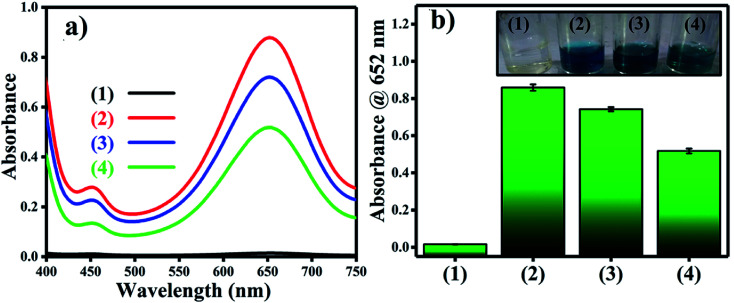
Validation of free radical species using UV/Vis. spectrophotometric analysis. (a) UV/Vis. responses of different reaction systems: (1) TMB + buffer, (2) TMB + buffer + Ag_3_PO_4_, (3) TMB + buffer + Ag_3_PO_4_ + IPA, TMB + buffer + Ag_3_PO_4_ + BQ. (b) Absorbances at 652 nm for the corresponding reaction systems. The inset of Fig. 2b shows the corresponding color changes of the different reaction systems.

Reactive oxygen species (ROS) were investigated by introducing iso-propyl alcohol (IPA) as an OH˙ scavenger and *p*-benzoquinone (BQ) as a superoxide radical 
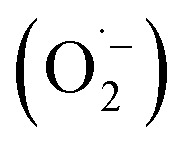
 scavenger. Fig. 11 shows the effects of the scavengers on the TMB oxidation process under all optimal conditions. Fig. 11a indicates the UV/Vis. spectroscopic results of oxTMB in the presence and absence of IPA and BQ based on the absorbance at 652 nm, as shown in Fig. 11b. The oxidation of TMB was found to be ∼16.1% reduced in the presence of IPA (reaction system 3), while in the presence of BQ, a ∼40.64% reduction in TMB oxidation was recorded. As a result, much fewer OH˙ free radicals are being generated, but comparatively higher amounts of 
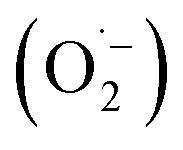
 are being generated in the reaction system through an *in situ* process; this helps to oxidize TMB and produce a blue product.^50^ These results were in good agreement with our previous report on free radical validation processes.^51^ These results have also been added to the manuscript and highlighted in red.

#### Thermocol alphabet imprinting sensing for chlorpyrifos

**Fig. 12:**
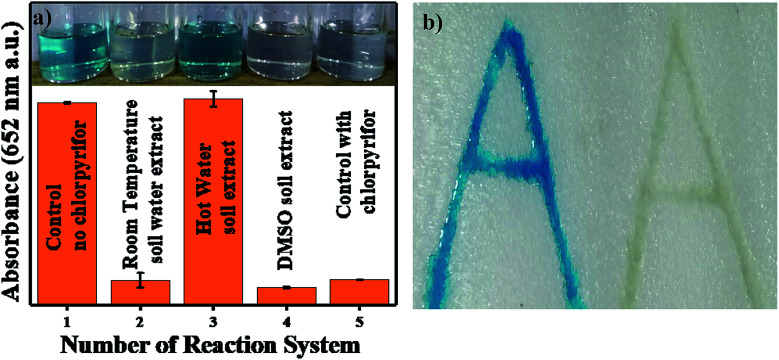
(a) TMB colour comparison of control and RT water, hot water and DMSO soil extracts. (b) Thermacol imprinted images without and with chylorpyrifos.

In Fig. 12a, the number of reaction systems was tested. The control experiment was performed in the absence of chlorpyrifos (set 1); set 2 contained water-washed aliquot + TMB, and set 3 contained hot water-washed aliquot + TMB, where the appearance of blue color shows that the expected impurities and 2 mg L^−1^ of chlorpyrifos were washed out by hot water washing. In DMSO, the soluble chlorpyrifos was extracted, and further spiking experiments were performed (set 4). A control experiment in the presence of standard chlorpyrifos was also performed (set 5). In this sensing method, the alphabet letter ‘A’ was imprinted twice on a thermocol sheet; both imprint volumes were filled with a solution of Ag_3_PO_4_ NPs dissolved in water, and the water was evaporated. Only one of the imprints was filled with chlorpyrifos to discriminate the difference in the presence and absence of chlorpyrifos, and both imprints were filled with TMB. Pale blue colour was observed in the chlorpyrifos-free imprint, and colour inhibition was observed with chlorpyrifos, as shown in Fig. 12b.

## 5. Conclusion

The present work demonstrates a novel method for single-step colorimetric sensing of chlorpyrifos. Ag_3_PO_4_ NPs were readily synthesized and characterized by different analytical tools, showing promising oxidase catalytic properties. The Ag_3_PO_4_ NPs were optimized in terms of concentration, temperature, time and pH for superior catalytic activity. The present sensing method shows comparatively low *K*_m_ values (the lower the *K*_m_ value, the higher the sensitivity) for standard and real soil samples for chlorpyrifos detection; this indicates that the Ag_3_PO_4_ nanozyme is more effective than the traditional HRP enzyme. The limit of detection (LOD) of standard chlorpyrifos was found to be about 9.97 ppm ≅ 10 ppm for standard samples. For real soil sample analysis, the Ag_3_PO_4_ NPs showed significant results for the detection of chlorpyrifos at different village sites through a spiking process. This thermocol imprinting sensing method is applicable for commercial use of the catalyst by applying this facile and inexpensive approach for sensing applications and towards device fabrication.

## Conflicts of interest

There are no conflicts to declare.

## Supplementary Material

RA-010-C9RA10719C-s001
