# Supplementary material for: Colorimetric sensing of chlorpyrifos through negative feedback inhibition of the catalytic activity of silver phosphate oxygenase nanozymes
Source: RSC Adv. 2020 Apr 1;10(22):13050–65. doi: 10.1039/c9ra10719c (PMC9051377; doi:10.1039/c9ra10719c)
Supplement: RA-010-C9RA10719C-s001 [file RA-010-C9RA10719C-s001.pdf]

Electronic Supplementary Material (ESI) for RSC Advances.
This journal is © The Royal Society of Chemistry 2020

Supplementary Information

Colorimetric sensing of Chlorpyrifos through Negative Feedback inhibition of Silver Phosphate oxygenase Nanozymes Catalytic activity

Amisha Kushwaha[#], Gajendar Singh[#] and Manu Sharma*[#]

Central University of Gujarat, Gandhinagar

Gujarat-382030

Corresponding Author: Dr. Manu Sharma

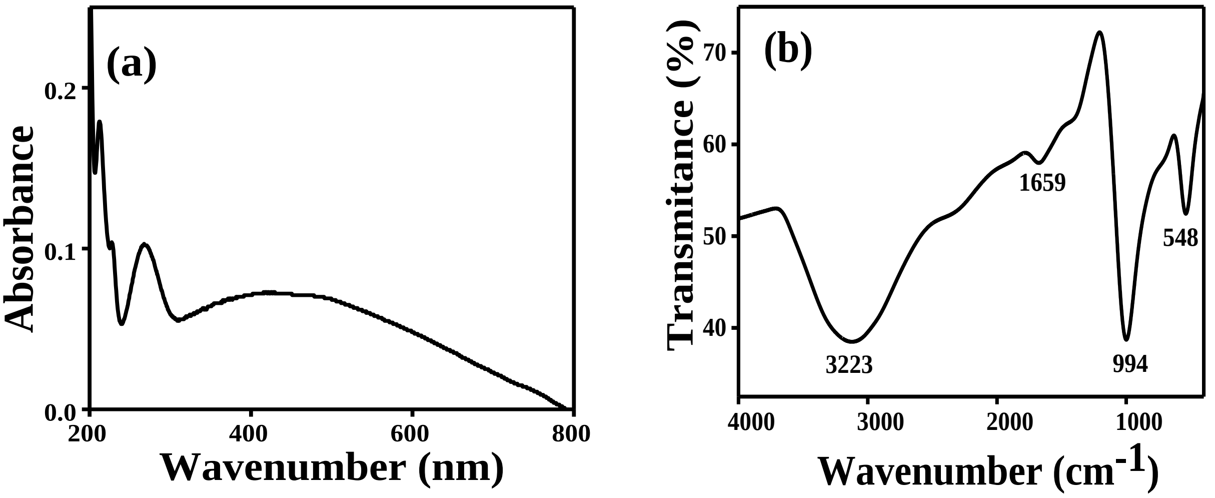

Figure S1:- (a) UV-Vis absorption spectra of Ag_3PO_4 NPs and (b) FTIR Plot of Ag_3PO_4 NPs

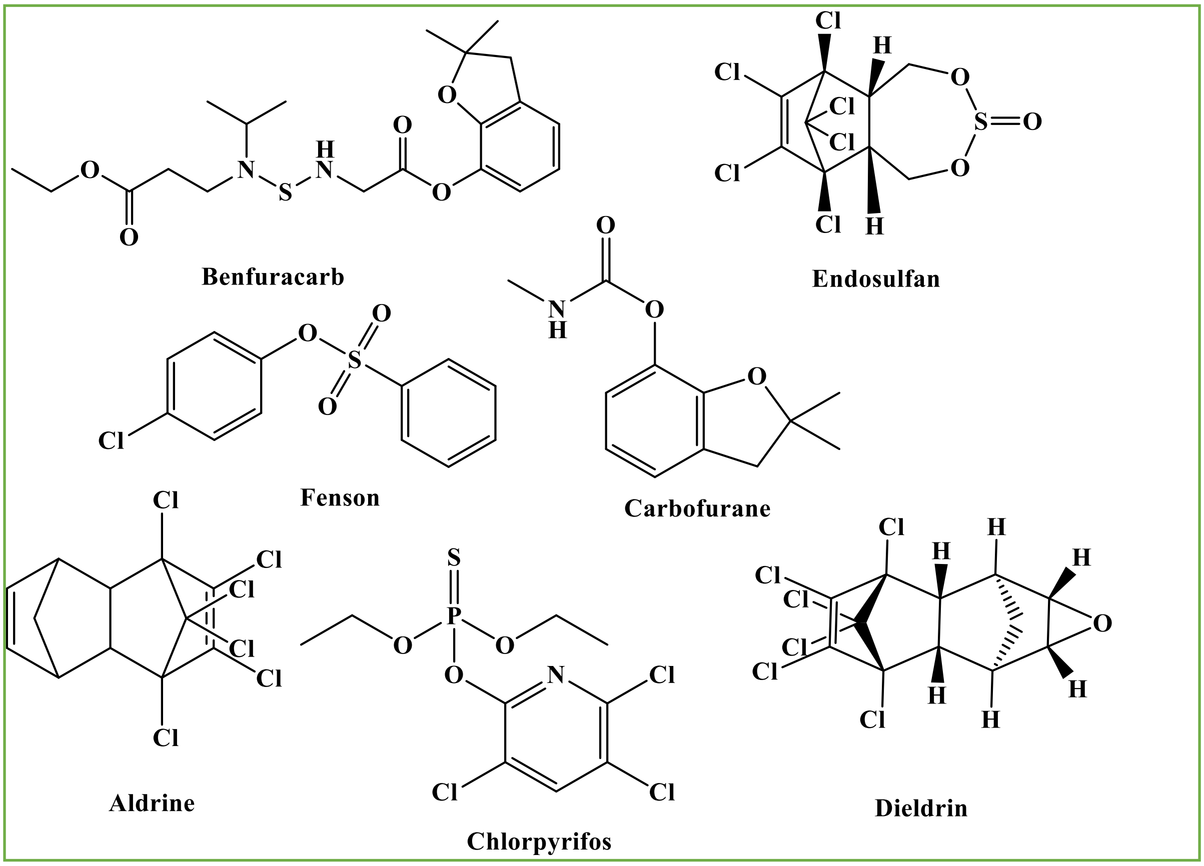

Figure S2:- All seven pesticide among these chlorpyrifor is selectively sensed and detected

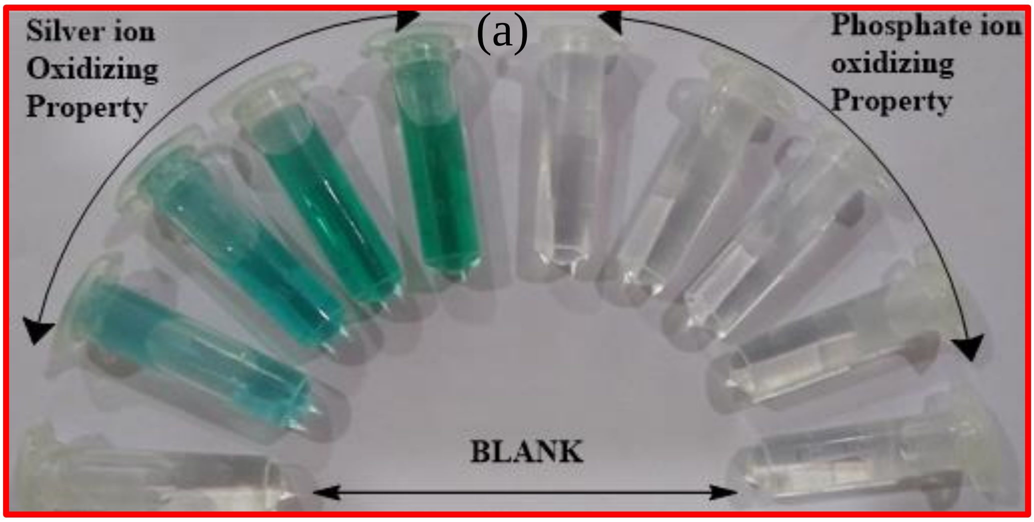

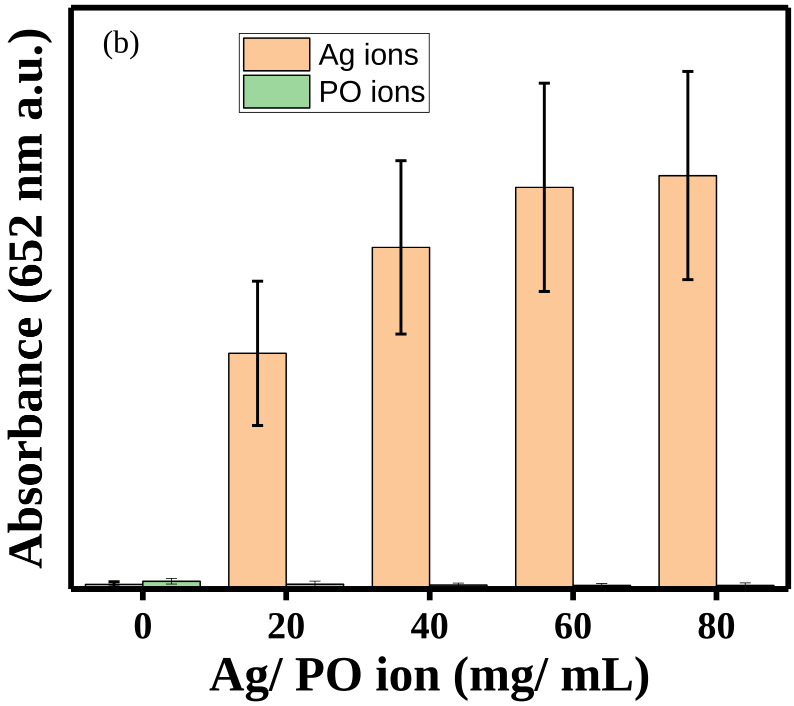

Figure S3:- (a) Digital picture for the oxTMB (blue colour) silver ion and phosphate ion oxidizing property with blank (b) Ag ion and PO ion graph for colorimetric sensing ability of ions

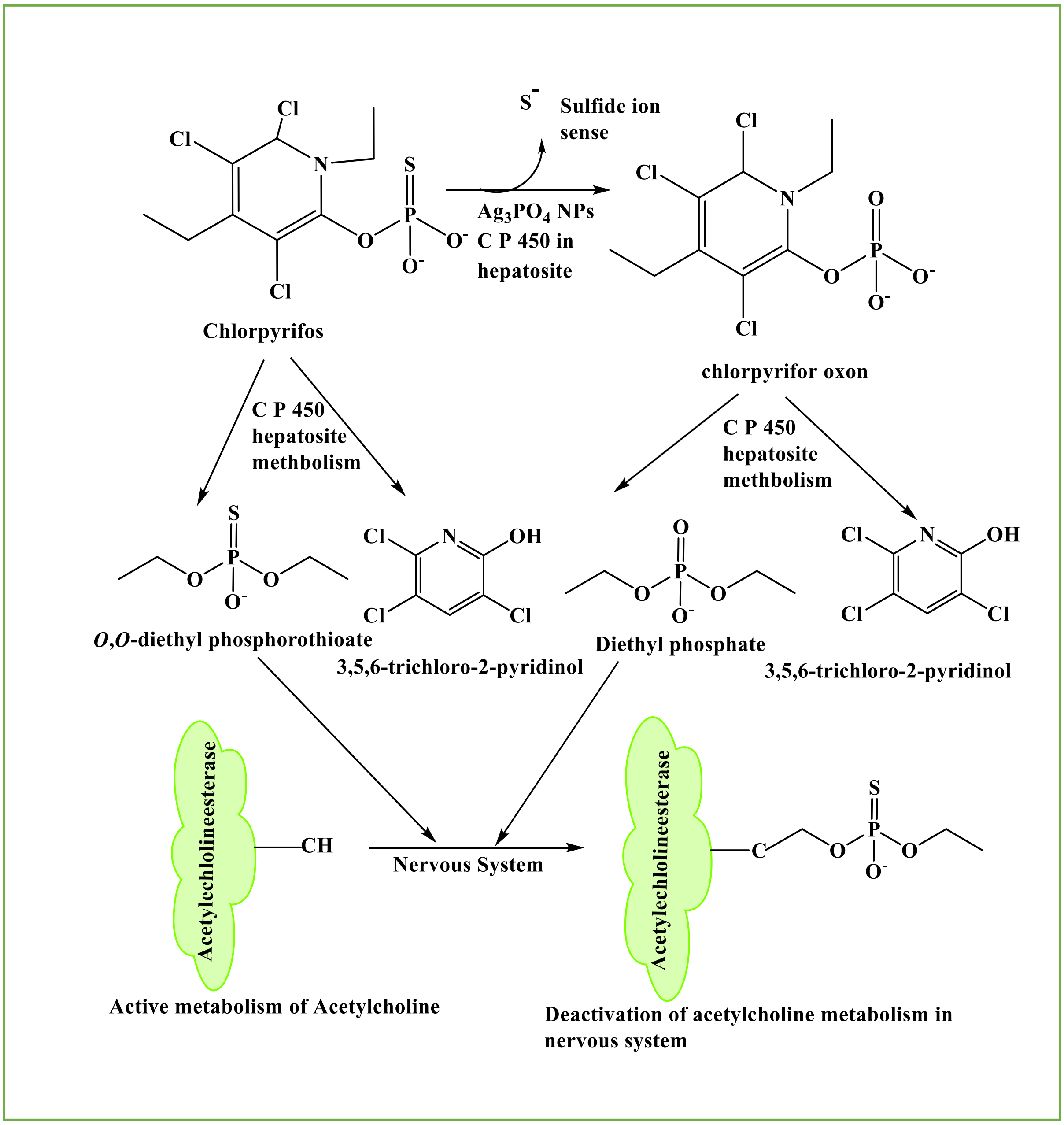

Figure S4:- Metabolism of chlorpyrifos in human liver (hepatocyte) through enzyme C P 450 (Cytochrome P 450) and inhibition of acetylcholinesterase to inhibit metabolism of acetylcholine in human nervous system throughout the body[1], [2], [3] .

References

1 A. L. Crane, K. Klein and J. R. Olson, *Xenobiotica*, 2012, **42**, 1255–1262.

2 V. Račáková, D. Jun, V. Opletalová and K. Kuča, *J. Appl. Biomed.*, 2006, **4**, 147–151.

3 A. L. Rathod and R. K. Garg, *J. Forensic Leg. Med.*, 2017, **47**, 29–34.
